# Levofloxacin HCl-Loaded Eudragit L-Based Solvent Exchange-Induced In Situ Forming Gel Using Monopropylene Glycol as a Solvent for Periodontitis Treatment

**DOI:** 10.3390/gels9070583

**Published:** 2023-07-18

**Authors:** Setthapong Senarat, Sarun Tuntarawongsa, Nutdanai Lertsuphotvanit, Catleya Rojviriya, Thawatchai Phaechamud, Takron Chantadee

**Affiliations:** 1 Programme of Pharmaceutical Engineering, Faculty of Pharmacy, Silpakorn University, Nakhon Pathom 73000, Thailand; senarat_s@silpakorn.edu; 2 Pharmaceutical Intellectual Center “Prachote Plengwittaya”, Faculty of Pharmacy, Silpakorn University, Nakhon Pathom 73000, Thailand; tuntarawongsa_s@su.ac.th; 3 Program of Pharmaceutical Technology, Department of Pharmaceutical Technology, Faculty of Pharmacy, Silpakorn University, Nakhon Pathom 73000, Thailand; lertsuphotvanit_n@silpakorn.edu; 4 Synchrotron Light Research Institute, Mueang District, Nakhon Ratchasima 30000, Thailand; catleya@slri.or.th; 5 Department of Industrial Pharmacy, Faculty of Pharmacy, Silpakorn University, Nakhon Pathom 73000, Thailand; 6 Natural Bioactive and Material for Health Promotion and Drug Delivery System Group (NBM), Faculty of Pharmacy, Silpakorn University, Nakhon Pathom 73000, Thailand; 7 Department of Pharmaceutical Sciences, Faculty of Pharmacy, Chiang Mai University, Chiang Mai 50200, Thailand; 8 Center of Excellent in Pharmaceutical Nanotechnology, Chiang Mai University, Chiang Mai 50200, Thailand

**Keywords:** Eudragit L, in situ forming gel, levofloxacin, monopropylene glycol, solvent exchange, periodontitis

## Abstract

Solvent exchange-induced in situ forming gel (ISG) is currently an appealing dosage form for periodontitis treatment via localized injection into the periodontal pocket. This study aims to apply Eudragit L and Eudragit S as matrix components of ISG by using monopropylene glycol as a solvent for loading levofloxacin HCl for periodontitis treatment. The influence of Eudragit concentration was investigated in terms of apparent viscosity, rheological behavior, injectability, gel-forming behavior, and mechanical properties. Eudragit L-based formulation presented less viscosity, was easier to inject, and could form more gel than Eudragit S-based ISG. Levofloxacin HCl-loading diminished the viscosity of Eudragit L-based formulation but did not significantly change the gel formation ability. Higher polymer loading increased viscosity, force-work of injectability, and hardness. SEM photographs and µCT images revealed their scaffold formation, which had a denser topographic structure and less porosity attained owing to higher polymer loading and less in vitro degradation. By tracking with fluorescence dyes, the interface interaction study revealed crucial information such as solvent movement ability and matrix formation of ISG. They prolonged the drug release for 14 days with fickian drug diffusion kinetics and increased the release amount above the MIC against test microbes. The 1% levofloxacin HCl and 15% Eudragit L dissolved in monopropylene glycol (LLM15) was a promising ISG because of its appropriate viscosity (3674.54 ± 188.03 cP) with Newtonian flow, acceptable gel formation and injectability (21.08 ± 1.38 N), hardness (33.81 ± 2.3 N) and prolonged drug release with efficient antimicrobial activities against *S. aureus* (ATCC 6538, 6532, and 25923), methicillin-resistant *S. aureus* (*MRSA*) (*S. aureus* ATCC 4430), *E. coli* ATCC 8739, *C. albicans* ATCC 10231, *P. gingivalis* ATCC 33277, and *A. actinomycetemcomitans* ATCC 29522; thus, it is the potential ISG formulation for periodontitis treatment by localized periodontal pocket injection.

## 1. Introduction

In the present era, approximately 20–50% of the world’s population is affected by periodontal diseases [1,2]. Among these individuals, more than 19.8% of adults and 12.2% of the elderly suffer from severe periodontitis, indicated by a periodontal pocket depth exceeding 6 mm [3]. This severe condition increases the risk of infection, inflammation, and tooth loss and negatively impacts the individual’s overall quality of life.

Periodontitis, a serious gum infection that destroys soft tissues and bone that supports the teeth, causes a periodontal pocket [4]. When periodontitis develops, pockets form as the inner gum pulls away from the teeth. These pockets collect debris and bacteria, which accelerates infection [5]. The periodontal pocket accumulates various bacteria, mainly pathogens like *Porphyromonas gingivalis* and *Aggregatibacter actinomycetemcomitans,* which have been recognized as the primary cause of advanced disease of periodontitis [6,7]. These pathogens are *Gram*-negative, facultative anaerobes, a nonmotile bacterium often detected in association with localized aggressive periodontitis, a severe infection of the periodontium [8]. Effective periodontal treatment involves the removal or inhibition of bacterial growth on tooth surfaces and within the crevicular pocket through methods like mechanical scaling, root planning, and medications. Antimicrobial agents, including chlorhexidine, tetracyclines (such as tetracycline, doxycycline, and minocycline), metronidazole, ciprofloxacin, vancomycin, and other antimicrobial drugs, are commonly used in periodontal treatment to eliminate infection [9,10]. However, the use of systemic antibiotics for periodontitis treatment necessitates large doses to achieve appropriate concentrations in the gingival crevicular fluid of the periodontal pocket, potentially leading to side effects and concerns about antibiotic resistance in other parts of the body and disrupting normal flora [11,12,13,14]. These limitations have prompted the development of local drug delivery systems for periodontal disease treatment. Implementing a controlled release system that delivers antimicrobial agents directly into the crevicular pocket is a more desirable strategy. Local drug delivery restricts the drug to the target site with minimal systemic uptake, allowing for lower doses to be effective and reducing or eliminating harmful side effects [15]. In this study, Eudragit L and Eudragit S were utilized as matrix components in an in situ forming gel (ISG) formulation by using monopropylene glycol as a solvent for loading levofloxacin HCl for periodontitis treatment. The development of worthy local drug delivery systems or devices attracted astonishing attention to overcome the restricted entry for eradication of the periodontopathic microbes in the deep periodontal pocket [16]. The in situ forming gel (ISG) system, induced by solvent exchange, has gained significant attention as a promising injectable drug delivery system for periodontal pocket administration. This is due to its simple administration process and uncomplicated fabrication, which involves the incorporation of a matrix-forming agent to regulate and localize drug release, while also providing effective antimicrobial activity [17]. The ISG system initially consists of a drug dissolved or dispersed in a polymer solution that thereafter solidifies via *a* water-solvent exchange mechanism from a fluid state into a drug-entrapped polymeric gel and matrix over time after injection and exposure to the aqueous fluid in periodontal pocket [17,18]. Atridox^®^ is a commercially marketed ISG containing D,L-lactic acid (PLA) and *N*-methyl-2-pyrrolidone (NMP) loaded with doxycycline hyclate for periodontitis treatment [19]. Doxycycline hyclate-incorporated ISGs for periodontitis treatment using ethyl cellulose and bleached shellac as the polymers have been reported recently [20]. Borneol, lauric acid, and some natural resins (such as propolis, benzoin, and rosin) are currently being applied as matrix-forming agents of ISG for modulating vancomycin HCL and doxycycline hyclate release for periodontitis treatment [21,22,23]. Eudragit RS has been employed as a polymer for the controlled release of doxycycline hyclate from ISG, in which the addition of peppermint oil and polyethylene glycol could modify drug release [24,25,26,27,28]. All the above-mentioned ISGs use NMP or dimethyl sulfoxide (DMSO) as solvents to dissolve drugs and polymers. The fluid properties of ISG solution state are dependent on Eudragit type and solvent, and therefore, the utilization of Eudragit and solvent in pharmaceutical dosage forms should be considered [29,30]. Increasing Eudragit L concentration in dimethyl sulfoxide (DMSO) has been shown to enhance the viscosity of its solution, and these polymeric solutions exhibited the potential to be developed into ISG for periodontal pocket drug delivery [31].

**Figure 1:**
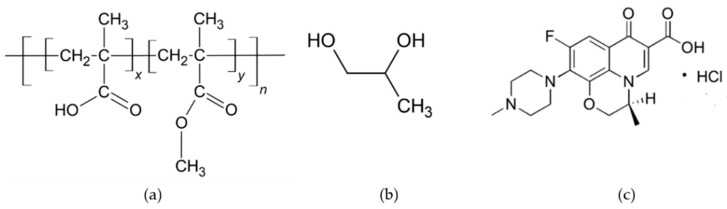
Chemical structure of Eudragit^®^ L and S 100 (**a**), monopropylene glycol (**b**), and levofloxacin HCl (**c**). The chemical structure of Eudragit^®^ L100 and Eudragit^®^ S100, where the ratio of x:y is 1:1 and 1:2, respectively.

Eudragit L and S (Figure 1a) are well-recognized as anionic polymers with enteric properties and are fabricated into various dosage forms including microspheres, micro-sponges, nanoparticles, matrix tablets, and coated tablets [32]. Eudragit L and S are known for their pH-dependent solubility. Eudragit L is insoluble in acidic environments but readily dissolving at pH levels above 6 [32], while Eudragit S dissolves at pH 7 or higher [33]. This characteristic is a key feature of the Eudragit line, as it allows for controlled drug release based on varying pH conditions [34]. Eudragit L has been used in the treatment of chronic enteritis as micro-sponges [35]. The strips that are prepared from Eudragit L/Eudragit S are used as intra-pocket delivery systems, and tetracycline HCl and clindamycin are the active agents that are contained within them [36]. These polymers have been utilized to produce nanoparticles that have been sanctioned for clinical application in Europe, Japan, and America. These nanoparticles are employed for the purpose of loading DNA plasmid and low molecular weight heparin [37]. Eudragit S is a negatively charged polymer employed for colonic drug delivery that dissolves at pH 7 or higher. Eudragit^®^ S transdermal film containing metoprolol tartrate may reduce first-pass drug metabolism [33]. Eudragits are commonly acknowledged as nontoxic and nonirritant materials. According to current research, the ingestion of polymethacrylates at a daily dosage of 2 mg per kilogram of body weight is considered to be safe for human consumption. The FDA Inactive Ingredients Guide lists Eudragit as one of its constituents [32]. ISG is employed as a polymer primarily from Eudragit RS and uses NMP as its solvent [24,25,26,27,28]. Nevertheless, the use of Eudragit L and Eudragit S in monopropylene glycol as the ISG system for periodontal treatment have not been investigated. These Eudragits dissolve at pH levels above 6, which might be easy to handle by providing a degradable matrix for drug release without the need for clinical removal.

Monopropylene glycol is also called propylene glycol, and its chemical formula C_3_H_8_O_2_ and structure are given in Figure 1b. Monopropylene glycol is a colorless, odorless, and water-soluble liquid. As an inert ingredient, the Food and Drug Administration (FDA) has given its approval for its use in a variety of products, including foods, tobacco products, and pharmaceuticals [38]. It was selected as a cosolvent in the preparation of in situ gel formulations of mitiglinide calcium for simultaneous extended delivery and enhanced drug bioavailability [39]. It has also been reported as a co-surfactant of an in situ microemulsion-gel in bio-adhesive hydroxypropyl methylcellulose films for zidovudine transdermal administration [40]. In this study, monopropylene glycol is used to dissolve Eudragit L- and Eudragit S-based ISGs since it is safe and has been used in the pharmaceutical field. Therefore, the present research’s conceptual framework is interested in applying Eudragit L and Eudragit S as polymers in monopropylene glycol of the ISG system to load antimicrobial agents for periodontitis treatment. Levofloxacin HCl was used as the antimicrobial drug in this study because of its effectiveness at low doses and broad-spectrum antibiotic activities [41]. To determine the appropriateness of using monopropylene glycol, compared to DMSO, we considered experimental results as well. Biocompatibility, on the other hand, is another matter that is based on the premise that their biocompatibility levels are acceptable, as the following reports: DMSO was used as a vehicle in various types of in situ formation systems, such as the in situ forming of implants containing osthole [42], risperidone, and paliperidone [43], and the in situ forming of microparticle loading montelukast sodium [44]. Intravesical instillation of 50% *w*/*w* DMSO (RIMSO-50^®^) is used as a therapeutic approach for interstitial cystitis. Because it did not cause any toxicity in the chondrocytes, the 7.813% *w/v* concentration of DMSO did not have a negative impact on the integrity of the articular cartilage [45]. Additionally, it was discovered that the utilization of a cryopreservation medium made up of fetal bovine serum and DMSO at a concentration of 5% was able to effectively preserve the colony formation and chondrogenic capabilities of synovial mesenchymal stem cells. Moreover, it was discovered that the utilization of a cryopreservation medium made up of fetal bovine serum and DMSO at a concentration of 5% was able to effectively preserve the colony formation and chondrogenic capabilities of synovial mesenchymal stem cells [46]. 

This study examined and analyzed the impact of varying concentrations of Eudragit on physicochemical properties, matrix-forming behavior, and antimicrobial activities. Furthermore, an investigation was conducted on the physicochemical properties of Eudragit ISG formulations that have been loaded with levofloxacin HCl. This investigation encompasses the apparent viscosity, rheology behavior, injectability, gel forming behavior, mechanical properties, matrix morphology, drug release, and antimicrobial activities of the prepared formulations.

## 2. Results and Discussion

### 2.1. Drug-Free Eudragit-Based ISG

#### 2.1.1. Physical Appearance, Viscosity and Rheology, Injectability, Contact Angle, and Mechanical Properties 

**Figure 2:**
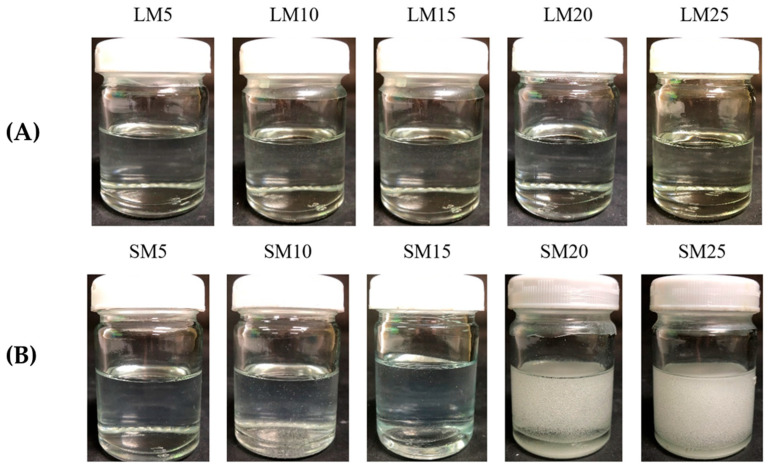
Physical appearance of Eudragit L (**A**) and Eudragit S (**B**)-based ISGs comprising different concentrations of polymers.

**Figure 3:**
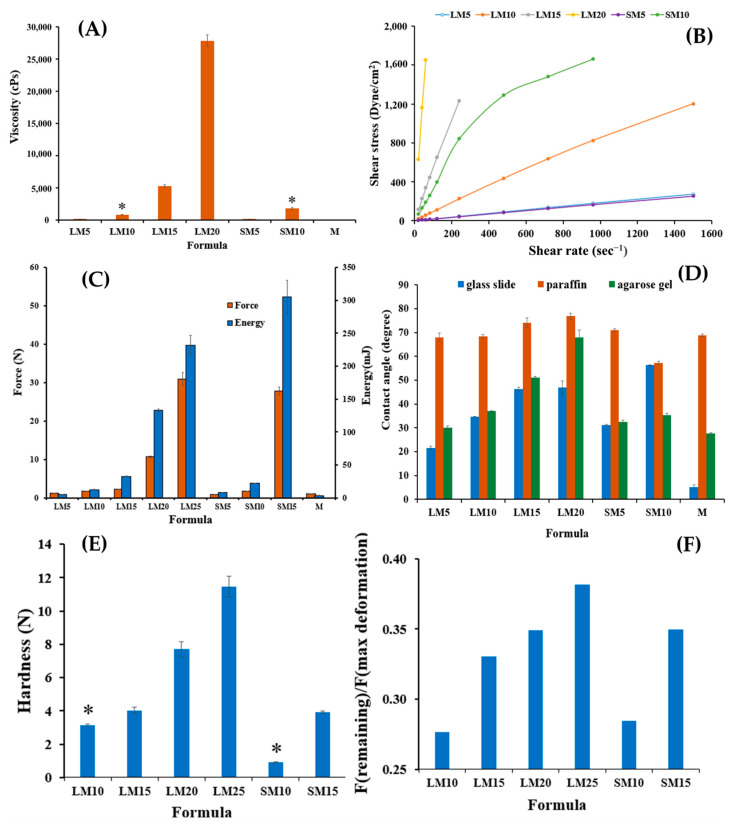
Viscosity (**A**); relationship between shear stress and shear rates (**B**); injection force and energy from injectability test (**C**); contact angle on different surfaces (**D**); hardness (**E**) and adhesiveness (**F**) properties from the mechanical test of Eudragit L and S-based ISG formulations at 25 °C. The data represent triplicates. The asterisk symbol indicates significant difference (*p* < 0.05).

Figure 2 depicts the effect of Eudragit L and S concentrations on the physical appearance of drug-free ISG formulations. All formulations were clear solutions, as shown in Figure 2B, except SM20 and SM25. This turbidity indicated a limitation in Eudragit S solubility in monopropylene glycol at higher concentrations than 15%. A slight yellowish color was observed in highly concentrated Eudragit L solutions. The viscosity of LM5, LM10, LM15, LM20, SM5, and SM10 formulations were 183.54 ± 2.00 cP, 820.02 ± 31.36 cP, 5292.68 ± 222.78 cP, 26214.29 ± 874.33 cP, 171.24 ± 3.16 cP, and 1884.15 ± 134.47 cP, respectively. The increasing Eudragit concentration resulted in high apparent viscosity of the ISG formulations (Figure 3A). Eudragit L ISG presented significantly less viscosity than Eudragit S ISG (*p* < 0.05). The Eudragit S formulations with polymer concentrations higher than 10% were excluded from viscosity measurement since they were too viscous for measuring. Because of the dominant effect of substance–solvent interaction over substance–substance interaction, a good vehicle for solubilizing the solute should reduce the viscosity of the mixture [47].

Figure 3B depicts the rheological characteristics of developed ISG formulations. The relationship between shear stress and shear rate was linear in the majority of formulations. Thus, the developed formulations displayed Newtonian flow characteristics. Consistent with prior study on the Newtonian behavior of Eudragit RS-based ISG, these results demonstrated the ISG’s Newtonian behavior [21]. The increased 3D network formation within Eudragit molecules can result in a significant increase in the viscosity of these formulations [48,49]. In spite of this, the SM10 curve went to a lower shear stress value, showing pseudoplastic flow behavior. Changing the number of molecular entanglements could affect the rheology and structure of entangled polymer systems [50]. Their Newtonian and pseudoplastic flow characteristics have been deemed suitable for injectable dosage forms because an injection through a needle is permissible after providing a compression force to the syringe plunger to eject the fluid through the stainless needle by a physician or dentist [49]. Eudragit L solution dissolved in monopropylene glycol exhibited less viscosity with Newtonian flow, making them more suitable for administration with injection than Eudragit S solution.

The administration of ISG should be simple and painless when using the appropriate needle and syringe. Localized ISGs for periodontitis treatment are designed for periodontal pocket delivery with an injection; thus, the developed fluid formulation should be easily expelled from the needle for less pain for the patient [51]. Therefore, it is vital to evaluate injectability, which refers to the amount of force required to expel the product through the needle. The maximum force for injectability of M, LM5, LM10, LM15, LM20, LM25 SM5, SM10, and SM15 formulations were 1.02 ± 0.04 N, 1.24 ± 0.07 N, 1.79 ± 0.05 N, 2.33 ± 0.02 N, 10.66 ± 0.30 N, 30.87 ± 1.68 N, 0.94 ± 0.02 N, 1.71 ± 0.01 N, and 27.74 ± 1.04 N, respectively. The low values of force and work of expulsion indicate ease of injectability. Figure 3C displays the force and work from the injectability determination of the test solutions. Their values increased with Eudragit concentration and corresponded to their apparent viscosity, as previously described. Work of expulsion for SM10 and SM15 were 20.22 ± 0.55 mJ and 305.05 ± 24.98 mJ, respectively. The notable shooting values were seen in a formulation comprising more than 10% of these polymers, especially for Eudragit S solution indicating that they had to be applied with a higher force and energy to expel from the needle. These results revealed that the kind and concentration of Eudragit had a significant impact on the needed injection force and energy. Nonetheless, the results demonstrated that the majority of ISG formulations required a low injection force (50 N). In practice, the total expulsive work required to inject each solution was less than 50 N.mm, suggesting that they met the injection criterion [51,52]. The addition of peppermint oil and polyethylene glycol (PEG) 1500 enhanced the injection performance of drug-free and doxycycline hyclate-loaded Eudragit RS ISG [27,28]. In comparison, the hydrophobic nature of peppermint oil provoked more ease of injection than PEG 1500 for the role of additives, such as peppermint oil and PEG 1500 on ISG [27,28]. The lubricating effect of oil incorporation on the ease of injection of in situ forming systems has been aforementioned [18,53]. For instance, the force and work of injectability of the rosin-based ISG formulations tended to reduce significantly after the addition of lime peel oil due to the lubricating nature of this oil [54]. Nonetheless, the ease of injection of Eudragit L correlated mainly to its lower viscosity. The molecule of polymeric material in solutions expresses the different chain configuration from its different affinity with the used solvents [55].

The contact angle could indicate the wettability or spreadability of that liquid on the test surface. The contact angle of monopropylene glycol was lowest on the glass slide (5.08 ± 1.05 degree) (as seen in Figure 3D). The contact angles on glass slide of LM5,LM10, and LM15 were 21.46 ± 0.75 degree, 34.70 ± 0.10 degree, and 46.27 ± 0.72 degree, respectively. Increasing contact angle on three surfaces was evident with polymer concentration dependence of Eudragit solutions due to the higher viscosity retardation of the spreadability of sample droplets on these three surfaces [21]. The agarose model (periodontal mimic) was employed to simulate the gum surface which provided small amount of simulated saliva fluid (PBS) continuously. Since it was found that the choice of release medium, such as water, phosphate buffer, or horse serum, did not have a big effect on phase separation, water uptake rates, or the morphology of the gel [56], PBS was used in this model instead of saliva fluid to reduce the number of variables.

The surface of agarose gel fabricated comprising PBS pH 6.8 was employed to mimic a surface inside the periodontal pocket. The contact angle of Eudragit-based ISGs was found to be notably higher than that of solvent onto this agarose surface (LM10; 36.97 ± 0.22 degree, LM15; 50.98 ± 0.58 degree, LM20; 67.98 ± 3.18 degree) especially higher than on the glass slide, indicating in situ transformation from solution to gel or solid-like Eudragit matrix provoking via a solvent exchange mechanism between monopropylene glycol in the formulation and aqueous solution in prepared agarose gels [18,53,54]. These Eudragit matrices resulting from solvent exchange diminished the spreadability of the formulations [47]. SM10’s high viscosity may hinder solvent exchange, and its contact angle on agarose gel was smaller than on the glass slide. Even though they had a high contact angle, their results were less than 90 degrees, indicating adequate wettability on test surfaces [57,58].

Typically, the obtained ISG matrix after solvent exchange should remain for fitting the periodontal pocket and resist the jaw’s motion while retaining its shape [59,60]. Figure 3E,F display the mechanical properties presented as hardness and F remaining/F max deformation. The ratio “F remaining/F max deformation” is used to assess the specimen’s elasticity/plasticity. A high value indicates high elasticity, while a low value indicates high plasticity [61]. These mechanical properties, especially the adhesive quality of transformed Eudragit ISG matrices, were designed to check the possibility of protecting undesirable slipping out of this dosage form from the periodontal pocket [58,62]. The introduction of Eudragits raised the hardness of the obtained matrix (LM10; 3.15 ± 0.08 N, LM15; 4.03 ± 0.19 N, LM20; 7.70 ± 0.45 N) owing to a greater polymer mass in the matrix. In comparison, the hardness and ratio of F remaining/F max deformation of Eudragit L-based ISGs were apparently and significantly higher than those of Eudragit S-based ISGs at the same concentration (*p* < 0.05). The ratio value of the matrix was significantly less than one, which indicated that the system’s behavior was close to plastic deformation. This meant that the system was unable to change its inner structure during the holding time permanently and was likely adaptable in terms of its geometry to dynamic changes in periodontal pocket size and shape with time [61]. This finding corresponds with the previously reported matrix from bleached shellac-based ISG, which was more likely plastic deformation or able to adapt its geometry to dynamic changes [60]. All of the Eudragit-based ISGs displayed adhesive strength properties against the texture analyzer probe, and all of the obtained gels remained attached to the agarose basement throughout the texture analyzer probe pullback.

#### 2.1.2. Gel Formation of Eudragit-Based ISGs

**Figure 4:**
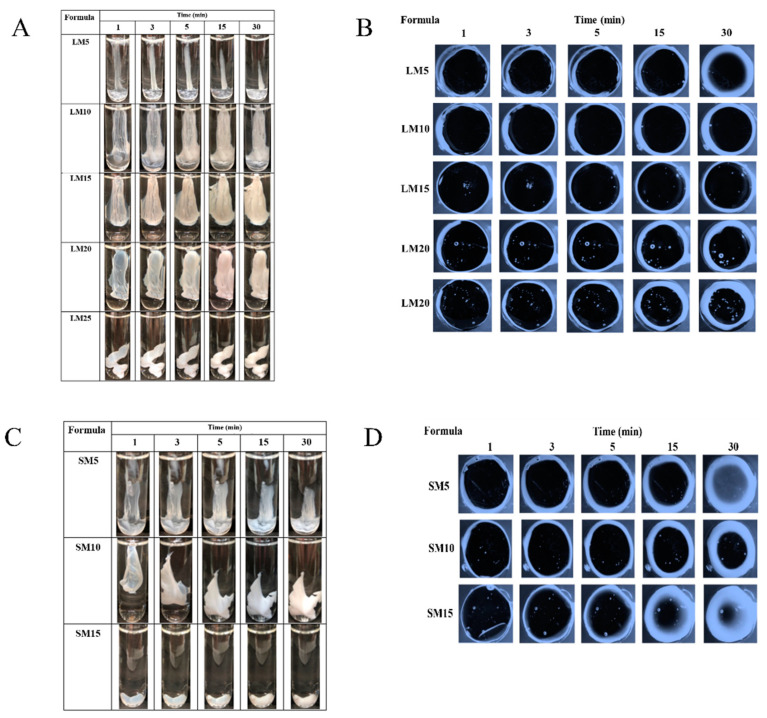
Gel formation after injection into PBS (**A**) and agarose well (**B**) of Eudragit L-based ISG formulations and gel formation after injection into PBS (**C**) and agarose well (**D**) of Eudragit S-based ISG formulations.

The phase transformation of all selected clear Eudragit-based solutions into a gel and subsequently an opaque matrix-like state over time are depicted in Figure 4A,C. The higher loading of these polymers promoted greater cloudy matrix formation due to the phase separation of Eudragits. From the results mentioned above, LM25 and SM15 exhibited more difficulty injecting through the needle and were more likely to stick in needles than other formulations due to their higher viscosity; therefore, they rapidly transformed into an opaque matrix and settled at the bottom of the PBS of the glass tube. During contact with PBS at pH 6.8, the formulation gradually incurred a solvent exchange, and the outer part initially changed into an opaque skin, while the inner gel phase continually transformed into an opaque solid polymeric matrix over time [49]. Therefore, more incorporation of matrix-forming agents accelerated the phase transformation of ISG. By comparison, there was no complete matrix formation of ISGs comprising ethyl cellulose, bleach shellac, and Eudragit RS of less than 10%, 20%, and 30% *w*/*w*, respectively, since all of them had no sufficient polymeric mass for continuing gel formation [20,24,26,63]. Figure 4B,D show a cross-section view of gel formation under the stereoscope using an agarose well to simulate the periodontal pocket. The phase separation of Eudragit resulted in an opaque layer at the rim of the agarose gel that expanded inward into the formulation. LM15 and SM10 showed slow matrix formation since their dense matrix possibly performs as a barrier and retard solvent exchange. There was a dense network of Eudragit matrixes formed at the interface. It slowed down over time since the high tortuosity and low porosity of the former matrix impeded the diffusion of solvent and water [64]. Nevertheless, the high inner polymer loading of LM20, LM25, and SM15 was found to promote Eudragit matrix growth. The mechanistic of Eudragit matrix formation could be explained through solvent exchange, in which the phase separation of dissolved Eudragit was evident into an insoluble solid matrix [26]. Owing to less viscosity, ease of injection, and interesting gel formation, 10, 15, and 20% Eudragit L-based formulations were selected for loading levofloxacin HCl and further investigation as ISGs for a periodontal pocket delivery system.

### 2.2. Levofloxacin HCl-Loaded Eudragit-Based ISG

#### 2.2.1. Physical Appearance, Viscosity and Rheology, Injectability, Contact Angle, and Mechanical Properties 

**Figure 5:**
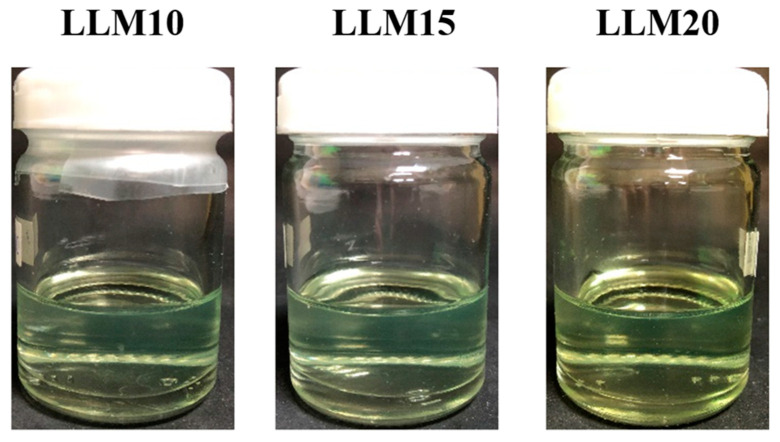
Physical appearance of levofloxacin HCl-loaded Eudragit L-based ISGs comprising different concentrations of polymer.

**Figure 6:**
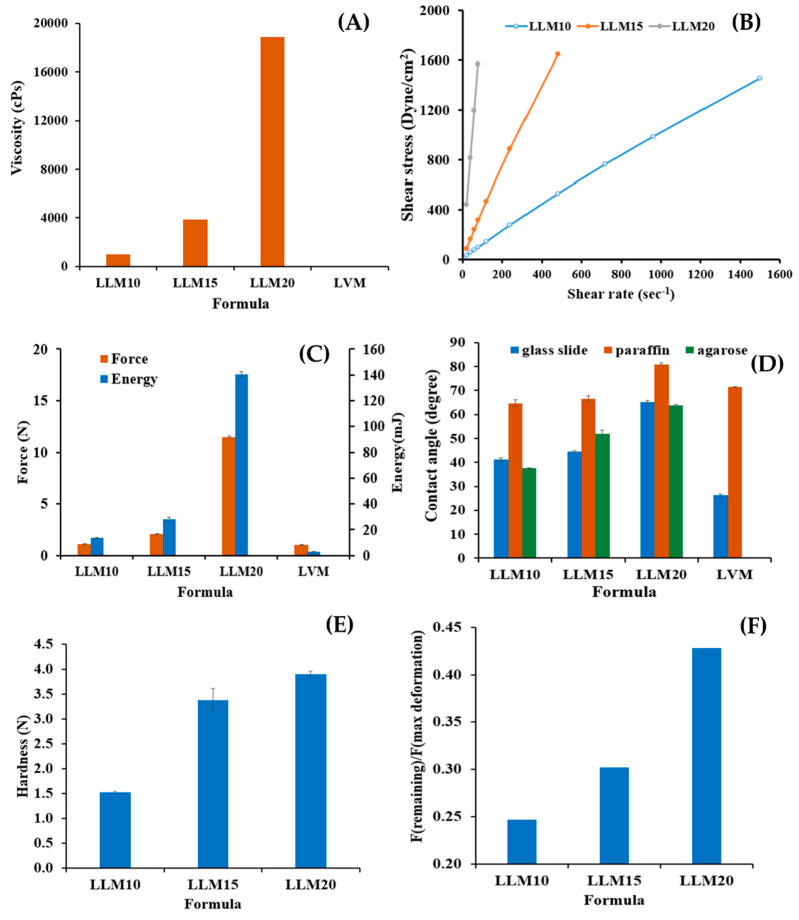
Viscosity (**A**); relationship between shear stress and shear rates (**B**); injection force and energy from injectability test (**C**); contact angle on different surfaces (**D**); hardness (**E**) and adhesiveness (**F**) properties from the mechanical test of levofloxacin HCl-loaded Eudragit L-based ISG formulations at 25 °C. The data are represented in triplicate.

The 10, 15, and 20% Eudragit L-based formulations were successfully prepared. Slightly yellowish solutions were obtained as presented in Figure 5. The viscosity was found to be lower after drug addition; nevertheless, increasing the Eudragit L concentration enhanced the viscosity (Figure 6A). The hydrophilic drug compound might impede the uncoiled Eudragit molecules and decrease the environmental viscosity. As presented in Figure 6B, all formulations retained Newtonian flow. The viscosity of LLM15 was 3674.54 ± 188.03 cPs. with the Newtonian flow. This flow behavior is considered to be suitable for injection dosage forms and is acceptable after injection [49]. The force and work of injection were enhanced with an increasing Eudragit L concentration (Figure 6C). The force of injection of LLM15 was 21.08 ± 1.38 N. The trend of contact angle was similar to that of drug-free Eudragit-based ISG formulations, denoting that the higher viscosity of systems due to Eudragit L addition enhanced the contact angle value by retarding the spreadability of the droplet. The higher contact angle of solutions, such as lauric acid and borneol dissolved in DMSO, has previously been reported and is related to their higher concentration and viscosity [21,65]. This increase in contact angle on the agarose gel surface was caused by the transformation of Eudragit L into a semisolid state, followed by the solvent exchange process and decreased droplet spreading [66]. Their contact angle values were less than 90°, indicating good wettability of these ISGs on test surfaces [58]. Practically, spreadability is needed for sufficient adhesiveness to protect the undesirable slipping out of the transformed ISG from the periodontal pocket [58,62].

Figure 6E,F are the mechanical properties of drug-loaded Eudragit L ISGs. The addition of more Eudragit L enhanced the hardness of the obtained drug-loaded matrix, whereas the ratio value of F remaining/F max deformation was less than one, indicating that the system’s behavior was similar to plastic deformation. These mechanical behaviors could inform their ability to adapt their geometry in the periodontal pocket, as aforementioned [61]. The hardness of the obtained LLM15 matrix was 33.81 ± 2.3 N. Some investigations reported that the addition of volatile oils improved the adhesion of the Rosin-ISG [67]. The rosin ISG with 10% cinnamon oil added had a higher adhesion property than that of 10% lime peel oil added, although formulations without the oil exhibited similar adhesion values [54]. And hence, the type of additive had a distinct impact on the adhesion properties of the ISG system.

#### 2.2.2. Gel Formation of Levofloxacin HCl-Loaded Eudragit-Based ISG

**Figure 7:**
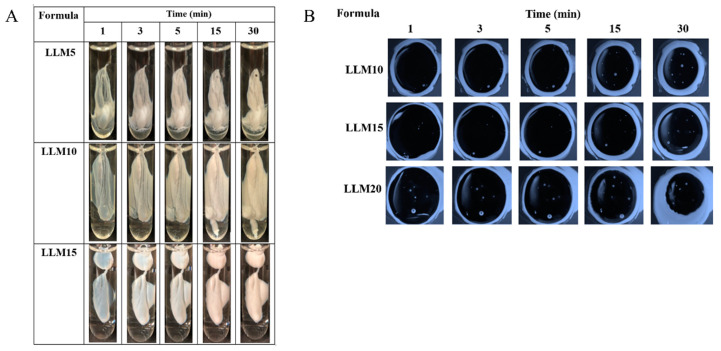
Gel formation after injection into PBS (**A**) and agarose well (**B**) of levofloxacin HCl-loaded Eudragit L-based ISG.

Figure 7A reveals that the greater cloudy matrix was formed after the injection drug-loaded with higher concentrate Eudragit L solutions or longer PBS exposure time. The phase separation of Eudragit L was due to its lower solubility in PBS than monopropylene glycol. Typically, high miscibility between the solvent and aqueous environment results in a rapid aqueous inward together with solvent outward, and thereafter a self forming process of aqueous insoluble matrix material was obtained [23,60].

A cross-section view of gel formation in Figure 7B indicated that a slower matrix formation of LLM15 corresponded with that of LM15 (Figure 4B) due to their dense matrix acting as a barrier for matrix growth by declining solvent exchange [64], and LLM20 could achieve a cloudier matrix. The addition of the drug did not affect the gel or matrix formation. In a similar vein, the addition of 1% vancomycin HCl had virtually no impact on the rate of matrix formation of borneol-ISG [21]. These test results confirmed that the injected drug-loaded Eudragit L-based ISG was capable of changing from a solution into a cloudy matrix instantly. The in situ process of Eudragit L-ISG presented after its contact with simulated periodontal fluid; thereafter, monopropylene glycol diffused outward simultaneously from the formulation with the entry of water, resulting in the hardening of cloudy Eudragit L gel into a solid-like matrix over time. The matrix formation of rosin-based ISGs induced by solvent exchange has been reported previously [18,54].

#### 2.2.3. Microscopic Interface Interaction 

**Figure 8:**
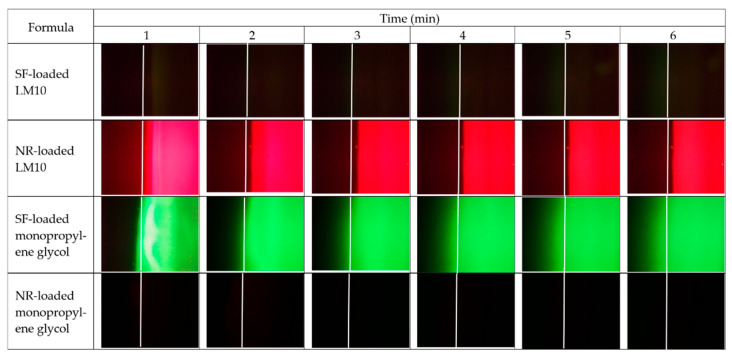
Interface interaction between noncolored agarose gel (the left side) against Eudragit L-based ISG formulation or mono propylene glycol containing SF or NR (the right side) at different time intervals under an inverted fluorescent microscope at a magnification of 400×.

**Figure 9:**
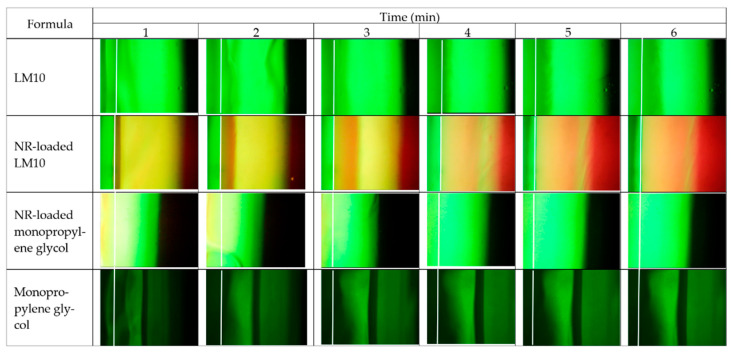
Interface interaction between sodium fluorescence-loaded agarose gel (the left side) against Eudragit L-based ISG formulation and monopropylene glycol without or containing NR (the right side) at different time intervals under the inverted fluorescent microscope at a magnification of 400×.

Figure 8 and Figure 9 display the movement of the fluorescent colors at the interface between agarose gel and formulation or solvent under a fluorescent microscope. LM10 was used as a model formulation in this study since its rather lower amount of polymer loading did not interfere with the emission of fluorescent dyes during its transformation into a gel or matrix-like structure. Figure 8 presents the color movement from Eudragit L-based ISG formulations or monopropylene glycol containing sodium fluorescein (SF) or nile red (NR) (right side) to noncolored agarose gel (left side). The white line indicates the interface between the right component and left agarose gel phase. SF is an orange-red, odorless powder that is freely soluble in water but only sparingly in alcohol [68]. Practically, SF absorbs blue light, with peak excitation occurring at wavelengths between 465 and 490 nm and resulting fluorescence occurring at yellow-green wavelengths between 520 and 530 nm [69]. When in neutral to an alkaline solution, it exhibits an intense yellow-green fluorescence under ultraviolet light [69]. The SF in the SF-loaded LM10 formulation did not express its green color in monopropylene glycol, but a dark band green was found at the interface, indicating that some SF diffused into the interface (Figure 8). However, the Eudragit L matrix formation impeded SFs further diffusion into agarose gel sufficiently to express its green color. Moreover, the water movement from agarose gel was insufficient to induce the emission of SF as bright green color generation in the formulation. Nile red is a hydrophobic probe that is intensely fluorescent [70]. Its fluorescence appears in organic solvents and preferentially dissolves in hydrophobic compounds, such as lipids, but is quenched in water [71]. NR-loaded LM10 exhibited red color from the NR emission initially dispersed into the agarose gel that might be caused by its diffusion along with monopropylene glycol, then it was absorbed onto the Eudragit L matrix; nonetheless, the mixing and diluting of water in agarose gel decreased NR solubility and diffused backward into the formulation, resulting in red color fading in agarose side while a darker red was seen in the formulation as more time passed. The occurred Eudragit L matrix was found as a white band at the interface at 1 min similar to the test with SF-loaded LM10. The higher intensity of fluorescence colors at equilibrium made it difficult to see this matrix band and its expansion. However, it seemed to prohibit SF and NR diffusion into agarose gel effectively. The fast diffusion of green color SF from monopropylene glycol into agarose was observed. Although SF did not express its color in monopropylene glycol, it abruptly presented a green color after being dissolved in a water phase. It was sudden green since the water from agarose (left side) faster migrate into the SF-loaded monopropylene glycol than that of migrate into SF-loaded LM10. As well as, NR-loaded systems, the rapid movement of water into the NR-loaded monopropylene glycol promptly reduced NR solubility and yielded in the absence of its color. While NR-loaded LM10 showed the red since the water influx was retarded and NR could present its emission color in the remained-monopropylene glycol. 

The microscopic interface interaction was also evaluated using SF-loaded agarose gel, as seen in Figure 9. The white line indicates the initial interface between two phases. The opaque expanding band occurred after SF-loaded agarose gel (left side) contacted with LM10 (right side), confirmed the matrix formation at the interface, and moved inward into the formulation as time passed, as also previously mentioned in the cross-section view of gel formation. These images also indicated that SF in formulation or solvent did not show its bright green color. For NR-loaded LM10, the initial Eudragit L matrix as a yellow band encompassed some NR at a narrow orange band near agarose gel and it diffused backward with solvent to accumulate in the formulation side and presented its red color more clearly. As aforementioned, the NR absorbed onto Eudragit L matrix could diffuse backward into the formulation, leading to the red color fading on the agarose side and a darker red in the formulation. The apparent green color was observed after SF-loaded agarose gel was contacted with NR-loaded monopropylene glycol, with the absence of clear red color. The initial NR red color appeared on the left side of the agarose due to its initial solvent diffusion. However, this solvent could be diluted after rapid mixing with water from the agarose gel. After adequate dilution, NR in the formulation was insoluble and could not express its color. The Eudragit L matrix of NR-loaded LM10 efficiently retarded the water movement into the formulation; therefore, there was enough solvent-maintained NR solubility to pronounce its red color. The rapid movement of SF with sufficient water from agarose into monopropylene glycol generated the brighter green color of this solvent over time. The back band should be the accumulation of monopropylene glycol at the interface with solvent font during contact time, and SF could not express its bright green color properly. Thus, applying fluorescence probes like SF and NR could provide crucial information such as solvent movement ability and matrix formation of ISG.

#### 2.2.4. Drug Content and Release of Levofloxacin HCl-Loaded Eudragit-Based ISGs

**Figure 10:**
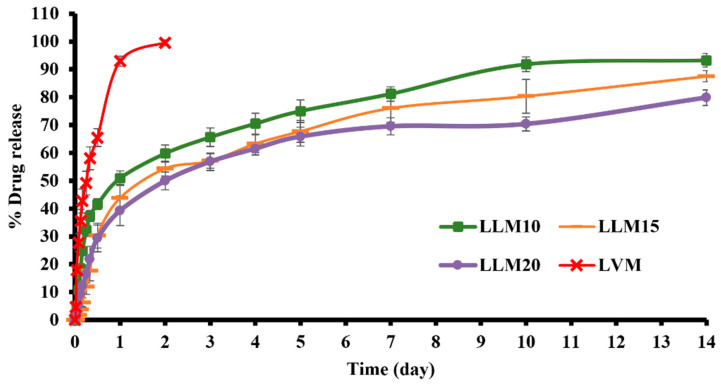
Drug release from Levofloxacin HCl-loaded Eudragit L-based ISG formulations (n = 3) (mean ± S.D).

The in vitro drug release was conducted in PBS at pH 6.8 to simulate the periodontal environment. The amount of drug content of LVM, LLM10, LLM15, and LLM20 samples were 102.52 ± 1.74, 100.85 ± 0.94, 98.77 ± 1.02, and 101.04 ± 0.86, respectively. After the preparation of the formulations, their amounts of drug content was determined before the in vitro drug release test using the cup method to mimic the environment of the periodontal pocket [54,67]. Previous researchers applied this method to study the drug release behavior of in situ forming drug delivery systems [20,21,63]. Figure 10 presents levofloxacin HCl release profiles from Eudragit L-based ISGs and LVM [control group]. There was a rapid levofloxacin HCl release from LVM, the control group which have no polymer. It released the drug completely in one day.

Sustainable drug release was attained from three developed ISGs. LLM20 had a more efficient prolongation of drug release than LLM15 and LLM10, respectively. They showed an initial burst drug release during the first day and gradually liberated the remaining drug during 1–14 days. The diffusion outward of monopropylene glycol into the release medium promoted a phase inversion and the formation of the beginning porous rubbery gel structure. These transformations occurred as a result of a burst release of drugs deposited on the surface layer. In the case of ISG using hydrophobic solvents such as triacetin and ethyl benzoate for dissolving PLGA, it exhibited slow gelation and significantly reduced the burst drug release [72]. This initial fast drug release behavior potentially promotes the high drug concentration to the therapeutic level above MIC for periodontitis treatment and, subsequently, the sustained release to maintain the drug level at the target site. Thermosensitive poloxamer 407 combined with chitosan gel showed an initial burst where about 60–70% of levofloxacin HCl was released within 6–7 h [73]. The thermosensitive gel, comprising 0.6% *w*/*v* gellan gum and 14% *w*/*v* pluronic F127, extended the release of levofloxacin HCl up to 3–4 h [74]. Therefore, these hydrophilic polymeric gels could insufficiently prolong the release of this drug. Doxycycline hyclate released from 15%, 25%, 30%, and 35% *w*/*w* Eudragit RS-loaded ISGs at 91%, 79%, 71%, and 68% at 54 h (2.25 days), respectively, since this polymer is a swellable, water-insoluble polymer and forms into different loose, swollen membrane structures in aqueous for drug release [24]. The commercial product for periodontitis treatment, such as Atridox^®^, uses 5% or 10% doxycycline hyclate as the antibacterial compound loaded into a 33.03% poly (D, L-lactide) solution sustained the drug release for at least 7 days [19,75]. In this study, levofloxacin HCl-loaded Eudragit L-based ISG formulations could control drug release for two weeks by utilizing a lower concentration of polymer than Atridox^®^. The developed ISGs present a fruitful alternative as a sustainable local drug periodontal pocket delivery system for improved patient compliance due to reduced drug administration frequency [16,61]. Moreover, from the literature review, the minimum inhibitory concentration (MIC) of levofloxacin HCl against *S*. *aureus*, *E*. *coli*, and *P*. *gingivalis* was 0.12 µg/mL [76], ≤0.12 µg/mL [77], and 8 µg/mL [78], respectively. The concentration of whole drug release from this study was 43.75 µg/mL (100% release); therefore, the 18.29% drug release or after around 4 h, all formulations could achieve the MIC of levofloxacin HCl against *P*. *gingivalis* and other test microbes.

The estimated parameters from the release profile fitting to the zero order, first order, Higuchi’s, and power law equations including regression coefficient (r^2^) value and model selection criteria (msc) are presented in Table 1. All of the release profiles were also consistent with the Korsmeyer–Peppas equation, with particularly high r^2^ and msc values. k is the constant incorporating structural and geometric characteristics of the device, and n is the release exponent revealing the drug release mechanism. Drug released from LVM fit well with a first-order equation (r^2^ and msc was 0.9744 and 3.3211, respectively) indicating drug concentration gradient-dependent behavior or a fraction of remaining drug in the solution. The r^2^ and msc and diffusion exponent value (n) obtained from the drug release profile of levofloxacin HCl-loaded Eudragit L-based ISGs close to 0.45 signified that they complied best with Higuchi’s equation. Thus, their kinetic of drug release was Fickian diffusion. Although their release profiles were well fitted with Higushi’s equation, increasing the polymer concentration significantly decreased the k value in Higushi, because the drug diffusion into the release medium was retarded by the denser Eudragit L matrix. The release of hydrophilic drugs from other polymer or fatty acid-based ISGs also mostly fitted well with Higuchi’s equation obeying Fickian diffusion kinetics [23,55].

#### 2.2.5. Scanning Electron Microscopy (SEM)

**Figure 11:**
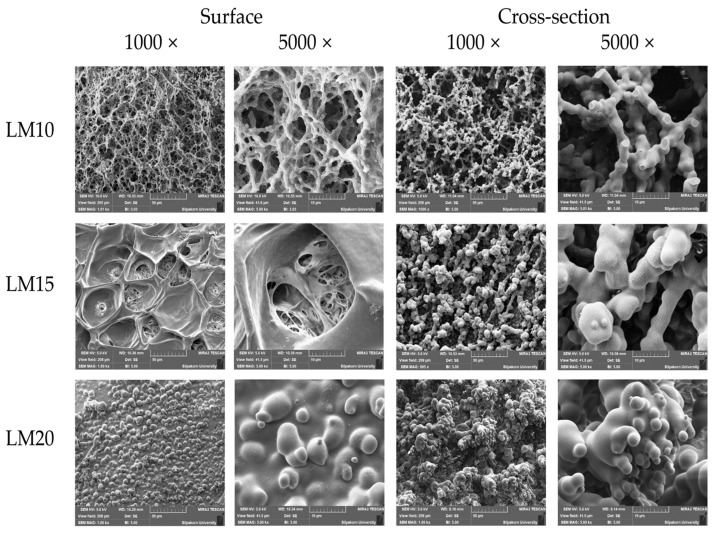
SEM images of surface and cross-section of freeze-dried remnant of Eudragit L-based ISG formulations after release test for 7 days at magnification of 1000× and 5000×.

Figure 11 presents the SEM photographs that exhibited the surface and cross-section topographies of dried remnants of drug-free Eudragit L-based ISGs (LM10, LM15, and LM20). The apparent scaffold topography was evident for LM10 both on the surface and cross-section. As a scaffold containing polymeric fibrous networks, this topography characteristic confirmed the readily solvent exchange promoting polymer separation. Nevertheless, the larger polymer loading initiated a denser surface while the inner larger fibrous occurred in the cross section-part. It seemed to have many spherical polymer particles connected and formed into a continuous fibrous network. The size of the void inside the structure was decreased with increasing polymer concentration. This obtained topographic characteristic was similar to that of the dried remnant of Eudragit RS-based ISGs as previously reported [24,27,28]. Eudragit L dissolves in the pH range of 5.5 to 6.0 and can be used to deliver medication to the ileum, while Eudragit S can provide release in the pH range of 7.0 and, hence, deliver the medication to the large intestine [32]. SEM photomicrograph of these developed ISGs, taken after the release study, presented that pores had occurred throughout the inner matrix with different densities depending on polymer content. As a result, the formation of both pores and scaffolds structure of ISGs signified the involvement of drug diffusion with solvent from a matrix or in situ forming scaffold into release medium [79]. The porosity and pore-connectivity were demonstrated to indicate how solvent migrated which subsequent construction of Eudragit scaffold and drug release. Nonetheless, the dissolution of Eudragit L in PBS 6.8 could occur continuously, and this process depended on the polymer content. The high-loading polymer content yielded a denser structure and lowered matrix dissolution. 

**Figure 12:**
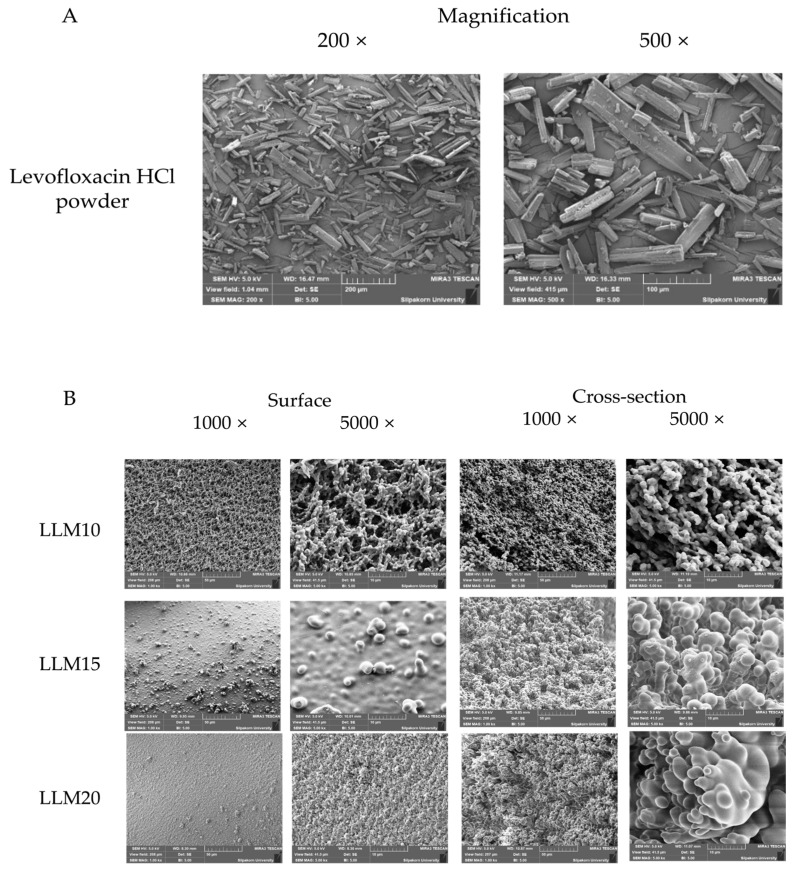
SEM images of levofloxacin HCl powder (**A**); surface and cross-section of a freeze-dried remnant of levofloxacin HCl-loaded Eudragit L-based ISG formulations at a magnification of 1000× and 5000× (**B**).

An SEM image of levofloxacin HCl powder revealed the crystalline rod-shaped-like particles (Figure 12A). The SEM photographs of the surface and cross-section topographies of levofloxacin HCl-loaded 10%, 15%, and 20% Eudragit L-based ISG remnants after 7 days of drug release are presented in Figure 12B. Because no drug crystals appeared in the remnants, the tiny amount of drug might disperse molecularly in a polymer matrix, and some dispersed drugs had already dissolved from the matrix. The enhanced concentration of Eudragit L remarkably promoted denser surface and inner topographies of the obtained polymer matrix. LLM20 achieved greater drug release prolongation than LLM15 and LLM10 due to a denser topographic matrix of ISG with higher polymer loading. By comparison, the porosity of drug-loaded ISG remnants was noticeably less than that of drug-free ISG remnants. The introduction of this hydrophilic drug could enhance the polarity of the formulation and might promote solvent exchange; therefore, the increased rate of phase separation of the polymer could induce a smaller fibrous formation. Furthermore, as aforementioned in Section 2.2.1, the inclusion of levofloxacin HCl decreased the viscosity of prepared ISGS. The nucleation and growth mechanism of the polymer in the dilute phase gives rise to the phase separation process, and the spinodal de-mixing that occurs during the membrane formation process is responsible for the interconnectivity that develops between the membranes [80]. Changes in solution viscosity caused by the addition of a hydrophilic drug could alter the phase separation rate, which ultimately causes the differences in matrix morphology as aforementioned for the addition of polyvinyl pyrrolidone (PVP) for producing polyethersulfone hollow fiber membranes [81]. The morphology of this membrane was evaluated as a function of the viscosity of the spinning solution, where the addition of PVP decreased the viscosity of the solution, favoring the high speed of precipitation and the formation of a specific type of pores similar to a finger in the membrane [81,82]. As shown in Figure 12B, the lower viscosity owing to drug loading that is mentioned in Section 2.2.1 may induce a more rapid phase separation of Eudragit L from solution and a less pronounced porosity and finger-like appearance of drug-loaded ISG remnants. It was reported that high polymeric concentration enhanced the gel resistance to aqueous influx and solvent outflux due to the less porous structure [83]. Hence, these SEM photographs emphasized the retardation of drug release of Eudragit L matrices, as distinguished previously in Section 2.2.4. The presented morphology of these remnants revealed the distinct porous structure demonstrating the involvement of solvent exchange as responsible mechanistic for modulating the drug release from Eudragit L-based ISG matrices. 

#### 2.2.6. In Vitro Degradation 

The behavior for in vitro degradation representing the % mass loss of three drug-loaded formulations is shown in Table 2. This mass loss is pronounced by the diffusion of the drug and monopropylene glycol as well as the dissolution of Eudragit L following an interchange with the release medium. The degradation of drug delivery systems could be described by their weight loss or molecular weight loss of carriers such as polymers [84]. ISG formulation containing a higher concentration of Eudragit L presented a less % mass loss, as shown in this table. On days 1 and 3, the three ISGs had significantly different values (*p* < 0.05), whereas that of LLM10 was significantly higher than that of LLM20 (*p* < 0.05) on days 5 and 7. The solvent exchange after immersion of these ISGs into the release medium initiated the formation of the porous matrix structure. Thus, the foremost mass loss from ISGs was owing to the diffusion of monopropylene glycol and also the drug release amount, which depended on the porosity and tortuosity of the Eudragit L matrices, as depicted and described in the previous SEM section. Typically, the degradability of polymeric structures such as Eudragit L is not due to enzyme or acidic conditions. Thus, the dominant mass loss that appeared in this investigation was from solvent leakage from the depot matrix and the gradual dissolution of Eudragit L mass. The complete mass loss was achieved at 14 days, as reported in Table 2, confirming that Eudragit L dissolution occurred gradually. The higher amount of matrix-forming agent enhanced the denser matrix structure and higher tortuosity, making it more difficult for monopropylene glycol and drug molecules to diffuse into the external medium. Thereafter, this phenomenon diminished in vitro degradation as well as prolongation of drug release. The steeper slope of the drug release profile during the early hours of this study likely occurred owing to the rapid release of the drug loaded onto or near the surface of Eudragit L matrix, followed by a sustained release of the drug distributed in the inner polymeric matrix. The composite material comprising polyurethane and Eudragit L as nanofiber fabricated by electrospinning technique exhibited adequate mechanical properties and in vitro cell biocompatibility, implying that the material is suitable for use in applications involving drug-eluting stent covers [34]. The incorporation of DNA plasmid for gene delivery and low molecular weight heparin delivery in nanoparticles fabricated by this group of polymers has been given the clinical go-ahead in the United States of America, Japan, and Europe. These nanoparticles have a low level of toxicity [37]. Eudragit L 100 is an anionic copolymer-based methacrylic acid and methyl methacrylic acid [32]. Its targeted drug release area is the jejunum, and it dissolves at a pH above 6 [32]. Eudragit L is a pH-dependent polymer soluble that dissolves continuously and slowly at a pH above 6. The amount of polymer present influenced the rate of degradation. Both the rate of degradation and the amount of degradation were increased due to the low polymer content. Because of this result, it was clear that this system would be useful for developing periodontal pocket drug delivery systems due to the fact that it degrades on its own in vitro over time.

#### 2.2.7. X-ray Computed Microtomography (μCT)

**Figure 13:**
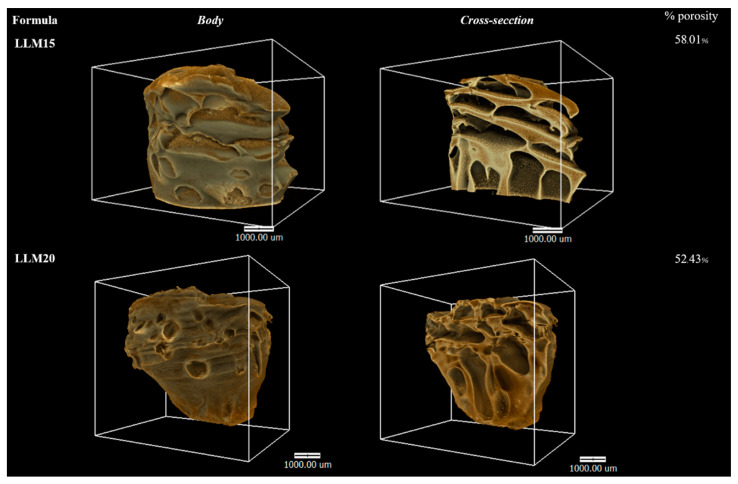
X-ray tomography image and %porosity using X-ray tomography of freeze-dried remnant after drug release test for 7 days of levofloxacin HCl-loaded Eudragit L-based ISG formulations.

μCT is used for interiorly visualizing a scanned physical solid object and obtaining information on its 3D geometries and properties. This method is based on the idea that the internal features of a solid object have variations in their density and/or atomic composition [85]. It has been applied to measure implant porosity and pore size in vitro [86]. The μCT images of LLM15 and LLM20 remnants obtained from a synchrontron light source are illustrated in Figure 13. The rather fragile LLM10 was not examined since it could not retain its feature for long period during assessment with synchrontron light. The 3D volume and the cross-section with the voids inside the LLM15 and LLM20 remnants are presented in 3D. The LLM20 remnant appears to have a less void structure than that of LLM15. Because of the striking contrast that exists primarily between solid phases and air, this method can be used to determine the porosity of the object that is the focus of the investigation [85]. Furthermore, the simultaneous analysis of the 3D structure showed that the %porosity of LLM20 was less than that of LLM15. This outcome was consistent with in vitro release behavior and topography (by SEM). Hence, the illustrated data from μCT was useful as supporting evidence. Previously, the μCT-imaging was applied for checking longitudinal quantification of degradation and intra-articular biocompatibility of hydrogel based on acyl-capped triblock copolymer poly[ɛ-caprolactone-co-lactide)-b-poly (ethylene glycol]-b-poly[ɛ-caprolactone-co-lactide] [87].

#### 2.2.8. Antimicrobial Activities

The inhibition zone diameter of solvents, drug-free, and levofloxacin HCl-loaded Eudragit L ISGs against *S. aureus* (ATCC 6538, 6532, and 25923), *methicillin-resistant S. aureus (MRSA)* (*S. aureus* ATCC 4430), *E. coli* ATCC 8739, *C. albicans* ATCC 10231*, P. gingivalis* ATCC 33277, and *A. actinomycetemcomitans* ATCC 29522 from antimicrobial activities test via cup agar diffusion method is shown in Table 3. These tested bacterial and fungal species are typically associated with periodontitis disease. The main obligate anaerobe pathogen bacteria of adult periodontitis have been identified as *Prophyromonas gingivalis* and *Aggregatibacter actinomycetemcomitans* [6,7]. In spite of this, more research needs to be carried out to determine the specific roles that each of these species plays, either on its own or in conjunction with other species, in the pathogenesis of periodontal breakdown. *Staphylococcus aureus* could be occasionally isolated from the periodontal pockets of patients with aggressive periodontitis, and *Escherichia coli* is sometimes declared as a microorganism in patients with periodontitis [88]. In contrast to the vast majority of other types of infections, all of the organisms that are thought to cause periodontal disease are native to the oral flora, *Candida albicans* courses of refractory periodontitis [89].

From this study, monopropylene glycol could slightly inhibit the growth of all test microbes, as presented in Table 3. It has been previously reported for antimicrobial effectiveness against three organisms, namely *S. mutans*, *E. faecalis*, and *E. coli*, and the bactericidal activity was at a concentration of 50%, 25%, and 50%, respectively [90]. Typically, organic solvents, such as monopropylene glycol, have the potential of disturbing lipids in the cell walls of microbes; thus, they could prohibit microbe growth. The drug-free Eudragit L-based ISG tended to inhibit microbe growth less than its solvent with polymer concentration dependence because the higher viscous and grater matrix formation noticeably impeded the movement of monopropylene glycol from ISG into inoculated agar media. Therefore the high polymeric concentration resulted in less antimicrobial activity. It was clearly confirmed since the LM15 and LM20 have no antimicrobial activity against *S. aureus* 25923, while solvent and LM10 showed inhibition zone. This role of the polymer in the retardation of solvent diffusion and antimicrobial activities has also been previously noticed in drug-free borneol-based ISGs [21]. 

Because of the absence of polymeric matrix and free drug spreading, the LVM solution acted as a control group with the largest diameter of the inhibition zone and was significantly larger than the drug-free formulations (*p* < 0.05) against almost all of the test microbes (Table 3). All drug-loaded ISGs demonstrated a significantly larger inhibition diameter than free-drug-loaded ISGs (*p* < 0.05) indicated the antimicrobial activity against all microbes, except the activity against *C. albicans.* Levofloxacin HCl have no activity on fungus [41]; therefore, LLM10, LLM15, and LLM20 were no different in the clear zone, compared to LM10-20. It was suggested that antifungal activity against C. albicans was mainly caused by the solvent because their clear zones were close to that of the solvent. Moreover, there was also a tendency for smaller inhibition clear zone with Eudragit L concentration dependent indicating retardation of the drug diffusion into inoculated media. These obtained results conformed with the viscosity of ISGs and their drug-release behaviors. In addition, all of the developed drug-loaded ISGs could inhibit *S. aureus* 25923. Hence, the Eudragit L matrices after phase transformation delayed drug movement with sustained drug release and decreased inhibition zone diameter. Some higher polymer-loaded ISGs, such as LLM15 and LLM20, had a significantly smaller inhibition zone diameter than LVM (*p* < 0.05), as presented in Table 3. Similar results were formerly obtained for the matrices that occurred from ISG phase transition fabricated using borneol [21], natural resins [18,22,54], cholesterol [91], polymers [53,92], and saturated fatty acids [23] as the matrix-forming agents of in situ forming systems. The inhibition zone diameter of drug-loaded formulations against *A. actinomycetemcomitans* ATCC 29,522 was larger than 40 mm; therefore, it could not observe and compare the efficacy between formulations. Nonetheless, this result demonstrated that they potently inhibited this pathogen. Therefore, the effective levofloxacin HCl ISG emerged as an attractive localized dosage form for periodontitis treatment in a controlled drug-release manner. Basically, the use of medicated in situ forming gel exhibited better clinical outcomes than scaling and root planning alone, which could be associated with the presence of monopropylene glycol which has antimicrobial activities but also performs as a good vehicle for ISG to deliver the drug into the periodontal pocket. LLM15 exhibited an appropriate viscosity with acceptable injectability and still retained its scaffold structure, as seen in μCT-imaging and SEM. Moreover, this ISG sustained the release of the drug above the MIC against the main pathogen, such as *P. gingivalis*. Thus, LLM15 emerged as the promising ISG formulation for periodontitis treatment. Eudragit L and monopropylene glycol are both safe and non-toxic. Although the safety data and applications of these compounds indicate the probability of both substances for injectable dosage forms, their safety and therapeutic efficacy of developed LLM15 ISG should be further determined in a clinical experiment.

#### 2.2.9. FOURIER Transform Infrared Spectroscopy (FTIR)

**Figure 14:**
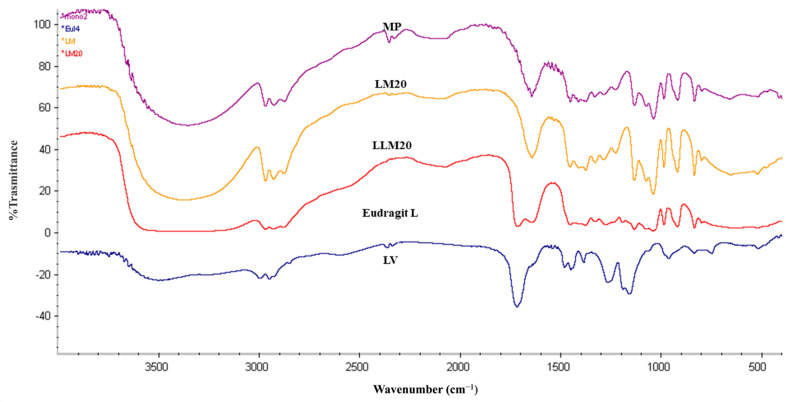
FTIR spectra of MP, LVM, LM20, LLM20, Eudragit L (raw material).

The analysis of the intact MP using Fourier transform infrared (FTIR) spectra revealed highly pronounced and distinct bands at 1650 cm^−1^ and 3000 cm^−1^. On the other hand, Eudragit L demonstrated the presence of a carbonyl band (C=O) between the wavelengths of 1720 and 1710 cm^−1^. There was no discernible difference in the peaks of either LM20 or LLM20, indicating that their trends were identical to those of the unaltered levofloxacin HCl (Figure 14). As a direct result of this, the conclusion that there was no interaction between Eudragit L and the solvent and LV was confirmed by the findings.

## 3. Conclusions 

We attempted to apply monopropylene glycol as a solvent to dissolve Eudragit L and Eudragit S, as well as levofloxacin HCl of solvent exchange-induced ISG. Eudragit L-based formulation in monopropylene glycol was selected for drug incorporation and further investigation as ISG for a periodontal pocket delivery system due to its good physical characteristics, including less viscosity, and more ease of injection with gel formation ability. The phase transition occurred from the solution of gel and matrix-like structures subsequently some time after ISG exposure to the aqueous environment of PBS and agarose gel. The diffusion of ISGs monopropylene glycol in water led to the hardening of cloudy Eudragit L gel into a solid-like matrix over time. Interface interaction study provided crucial information, such as solvent movement ability and matrix formation of ISG, by tracking fluorescence dyes. All developed levofloxacin HCl-loaded Eudragit L-based ISGs prolonged the drug release for 2 weeks via Fickian drug diffusion kinetics and the release amounts reached the MIC against test microbes. SEM and μCT images revealed their scaffold formation, which had a denser topographic structure and less porosity attained owing to higher polymer loading less in vitro degradation. LLM15 exhibited an appropriate viscosity, Newtonian flow, acceptable gel formation, and injectability, and prolonged drug release for 14 days with efficient antimicrobial activities against *S. aureus* (ATCC 6538, 6532, and 25923)*,* methicillin-resistant *S. aureus (MRSA) (S. aureus* ATCC 4430), *E. coli* ATCC 8739, *C. albicans* ATCC 10231, *P. gingivalis* ATCC 33277, and *A. actinomycetemcomitans* ATCC 29522; Therefore, it has the potential to be an ISG formulation used in the treatment of periodontitis. Even though there are safety data associated with a variety of medical applications of monopropylene glycol and Eudragit L, the newly developed LLM15 ISG is essential for further clinical experiments to evaluate the safety and therapeutic efficacy of the combination.

## 4. Materials and Methods

### 4.1. Materials

Both Eudragit^®^ L and Eudragit^®^ S came from EVONIK RohmGsmbH, which is located in Darmstadt, Germany. The antimicrobial agent levofloxacin HCl was generously provided by Siam Pharmaceutical Co., located in Bangkok, Thailand, and was put to use in this study. We visited P.C. Drug in Bangkok, Thailand, in order to purchase monopropylene glycol, which was then put to use as the solvent. During the investigation of the gel formation process, agarose with lot number H7014714 from Vivantis in Selangor Darul Ehsan, Malaysia, was utilized. For antimicrobial research, the following agars were utilized as media: sheep blood agar and chocolate agar (both from the Ministry of Public Health in Nonthaburi, Thailand), tryptic soy agar and tryptic soy broth (both from DifcoTM in Detroit, MI, USA). Sabouraud dextrose agar (SDA) and Sabouraud dextrose broth (SDB) (both manufactured by Difco in Detroit, MI, USA) were used in the antifungal test. Components of the phosphate-buffered saline solution included potassium dihydrogen orthophosphate (lot no. E23W60, Ajax Finechem, New South Wales, Australia) and sodium hydroxide (lot no. AF310204, Ajax Finechem, New South Wales, Australia). Both of these were obtained from Ajax Finechem (PBS). Test microbes, *S. aureus* ATCC 6538, *E. coli* ATCC 8739, *Candida albicans* ATCC 10231 (Department of Medical Sciences, Ministry of Public Health, Nonthaburi, Thailand), *P. gingivalis* ATCC 33277, and *A. actinomycetemcomitans* ATCC 29522 (MicroBiologics Inc., St. Cloud, MN, USA) were purchased from Thai Can Biotech Co. *P. gingivalis* ATCC 33277 and *A. actinomycetemcomitans* ATCC 29522 were two of the anaerobe bacteria that were cultured on sheep blood agar and chocolate agar, respectively, in this study. For the purpose of developing the two-dimensional structure of Eudragit L 100, ACD/ChemSketch was utilized. As a water-soluble fluorescent dye and a lipophilic fluorescence probe, respectively, sodium fluorescein (Carl Roth GmbH, Karlsruhe, Germany) and Nile red powder (SIGMA-Aldrich Chemie GmbH, Steinheim, Germany) were used for the interfacial interaction that occurred during phase transformation.

### 4.2. Preparation of In Situ Forming Gel

Both Eudragit L and S were separately dissolved in monopropylene glycol at a range of different concentrations over the course of an overnight stir session in an airtight container. In order to investigate the physical properties, systems were selected in which the components had completely dissolved, the liquid was transparent and flowable, and there was no formation of solid matter or gel. Table 4A,B detail the constituents that make up drug-free ISG. The polymers and levofloxacin HCl at a concentration of one percent were prepared using the aforementioned method. The components of the drug-loaded ISGs are outlined in Table 4C.

### 4.3. Viscosity and Rheology Characterization

The apparent viscosity values were measured using a viscometer with a cone-plate called the RM 100 CP2000 plus, which was manufactured by Lamy Rheology Instruments Company and is located in Champagne-au-Mont-d’Or, France. To carry out this measurement, a series of measurements at varying shear rates were taken at regular 15-s intervals during the equilibration time. This instrument was also used to test the rheological behavior of the substance. At room temperature, the shear stress of the formulations was determined by conducting the experiment at a variety of shear rates. The experiments were carried out three times to ensure accuracy.

### 4.4. Contact Angle

The goniometer (FTA 1000, First Ten Angstroms, Newark, CA, USA) was used to determine the contact angle of the formula on various surfaces, such as glass, paraffin, and agarose gel, at a time point of 5 s after a pump out rate of 1.9 L/s. The manufacturer of the goniometer is First Ten Angstroms. After that, an estimate of the contact angle was derived from the very first automatic image of a droplet that was taken in triplicate (n = 3).

### 4.5. Injectability 

Using the compression mode of a texture analyzer, a measurement of injectability was carried out in order to determine the precision of injection through the stainless needle (TA.XT plus, Stable Micro Systems, Godalming, UK). This apparatus was utilized to measure the pressure exerted during the injection of test liquid from a 1 mL syringe that was coupled to an 18-gauge needle. This device’s upper probe pressed the syringe plunger with a constant force of 0.1 N and a speed of 1.0 mm/second until it reached the base of the syringe barrel. After analyzing force-displacement profiles, the maximum injection force, along with its associated energy for injection, was logged. Three separate trials of these experiments were carried out.

### 4.6. Mechanical Properties

A texture analyzer was utilized in order to investigate the formulation’s mechanical properties after it had been prepared (TA.XT Plus, Stable Micro Systems Ltd., Godalming, UK). After setting with a complete phase transformation into Eudragit matrix for 72 h, the prepared 150 microliters formulation was added into 0.6 percent *w*/*w* agarose gel. This process took place for 72 h. After that, an analytical probe from the previously described instrument was lowered into the polymer matrix at a rate of 0.5 mm per second. After maintaining this position for sixty seconds, the probe was then moved upward at a rate of ten millimeters per second. The amount of force that was checked at the point where the probe was able to penetrate the polymer matrix to its maximum depth was indicated as the maximum deformation force or the hardness of the material. Additionally, the amount of force that was checked at the point where the probe was able to move upward between the surface of the sample and the probe was indicated as the adhesion force. The maximum deformation force, also known as F max deformation, is the force that is measured at the point where the probe has penetrated the specimen the farthest, whereas the remaining force, also known as F remaining, is a force that is measured after the specimen has been held for sixty seconds. A measure for the elasticity and plasticity of the specimen was the ratio “F remaining/F max deformation”. Values of elasticity that are high indicate a material with high elasticity, while values of elasticity that are low indicate a material with high plasticity [61]. The experiments were carried out in triplicate.

### 4.7. Gel Formation Study 

The apparent gel formation at macroscopic level was performed by injected the prepared ISG through an 18-gauge needle into PBS (pH 6.8) medium. The cloudy gel or matrix-like formation at various times was observed (1, 3, 5, 15, and 30 min). Moreover, another gel formation behavior investigation at macroscopic level was also investigated. The in situ formation in an agarose well was also investigated under a stereoscope. This experiment was set to simulate the change of formulation within a periodontal pocket. To begin, an agarose gel with a height of one centimeter was prepared by dissolving 0.6 percent agarose in PBS with a pH of 6.8. The gel was then poured onto Petri dishes for the setting process. The settle agarose was drilled using a stainless cylinder cup (7.6 mm diameter) to create an agarose well, the cylindrical well with a capacity of 300 microliters that simulated a periodontal pocket. For the purpose of testing, a prepared formulation containing 150 microliters was inserted into the well which is a simulated periodontal pocket. After being exposed to PBS from the agarose gel, the phase separation of Eudragit leads to the generation of a thicker, cloudier gel or matrix-like substance over time. Under a stereo microscope (SZX10, Olympus Corp., Tokyo, Japan), photographs of the morphological Eudragit gel or matrix were taken simultaneously at 1, 3, 5, 15, and 30 min. The software used for these photographs was the SZX10 Series software (Olympus Corp., Tokyo, Japan). 

### 4.8. Interfacial Phenomena of Formulation-Aqueous Phase

Both a plain agarose gel with a weight-to-volume ratio of 0.6 percent and an agarose gel loaded with 0.4 μg/mL of SF were prepared. The LL10M, 0.4 μg/mL SF-loaded MP, 0.4 μg/mL SF-loaded LL10M, 3 μg/mL nile red (NR) -loaded MP, and 3 μg/mL nile red (NR) -loaded L10M were prepared. The different levels of color intensity after the emission of these fluorescent dyes were the root cause of the difference in concentration between them. Based on preliminary inspections, it was found that their concentration could be clearly seen at an intensity that was sufficient to observe the change in color or movement. In order to investigate the interfacial interaction between an aqueous phase and a solvent or ISG formulation, the SF-free and SF-loaded agarose gels were prepared, and then 50 microliters of prepared test samples were dropped close to that agarose gel. The interface interaction was investigated using an inverted fluorescent microscope (TE-2000U, Nikon, Kaw, Japan) by capturing the image using a blue (B2A) filter with excitation at 450–490 nm for probing the green color of SF and using a green (G2A) filter with excitation of 510–560 nm for tracking the red color of NR. Both of these filters were used in conjunction with the appropriate wavelengths of illumination. 

### 4.9. Drug Content and In Vitro Drug Release Studies

Using a UV-Visible spectrophotometer (Cary 60 UV-Vis, Model G6860A, Agilent, Selangor, Malaysia), a standard curve was constructed to estimate the amount of drug in the LVM, LLM10, LLM15, and LLM20 samples (n = 6). The in vitro drug release behavior of levofloxacin HCl from a developed formulation comprising 10, 15, and 20 percent Eudragit L was undertaken to employ the cup method to mimic the drug liberation behavior from the periodontal pocket. The drug release profiles that were obtained were compared with those obtained from the control formulation, which consisted of levofloxacin HCl dissolved in monopropylene glycol at a concentration of one percent by weight. In a nutshell, 0.4 g of the refined dosage form was carefully placed into a cylindrical-shaped porcelain cup with 50 mL of PBS with a pH of 6.8 and heated to 37 degrees Celsius (diameter of 1 cm and higher of 1.2 cm). After that, it was placed in a rotation shaker (Model NB-205, N-Biotek, Gyeonggi-do, Republic of Korea) for 14 days at a temperature of 37 degrees Celsius and a rotational speed of 50 revolutions per minute. After that, 5 mL of release fluid was taken, and it was subsequently replaced with 5 mL of fresh PBS. The results of the study on the release of levofloxacin HCl were converted into a percentage of the cumulative drug amount using a UV-Visible spectrophotometer (Cary 60 UV-Vis, Model G6860A, Agilent, Selangor, Malaysia). The experiments were carried out three times to ensure accuracy.

Their drug release data were then fitted with a variety of mathematical models, such as zero order, first order, Higuchi’s, and Korsmeyer–Peppas models, using the DD-Solver software application, which is an add-in program for Microsoft Excel. This enabled the researchers to determine the kinetics of drug release (Microsoft Corporation, Redmond, WA, USA). The estimated parameters, in particular the r^2^ and model selection criteria (msc) values, were reported. Additionally, the release exponent (n-value) from the Korsmeyer–Peppas equation was used to indicate the mechanism of drug release. The following equations were used for mathematical model [93,94,95]. The zero-order model: the following equation indicates the release rate of the drug remains constant over time, independent of the drug concentration.

(1) $$Q_{t}=Q_{0}+k_{0}t$$

where Q_t_ is the total amount of drug release at given time (t), Q_0_ is initial drug release, k_0_ is the release rate constant, and t is the given time. 

The first-order model is another commonly employed mathematical model for describing drug release behavior. It assumes that the release rate is proportional to the remaining drug concentration as following equation.

(2) $$\log Q_{1}=\log Q_{0}+\frac{k_{1}t}{2.303}$$

where $Q_{1}$ is the amount of active agent released on time (t), $Q_{0}$ is the initial amount of drug dissolved, and $k_{1}$ is the first-order constant.

The Higuchi model assumes that drug release from polymer matrix systems is solely diffusion-controlled and that the drug is uniformly distributed in non-degradable planar systems. The Higuchi’s model is given below.

(3) $$Q_{t}=k\sqrt{t}$$

where Q_t_ is the total amount of drug release at given time (t), k is the release rate constant, and t is given time.

The Korsmeyer–Peppas model is widely used to assess the release mechanism. It incorporates a parameter ‘n’ to characterize the release mechanism, with values indicating different release kinetics. For swelling system, Fickian diffusion is indicated when the value of n is close to 0.45. If the value exceeds 0.45 but is lower than 0.89, this indicates non-Fickian diffusion or anomalous transport, and case II transport is indicated when n is close to 0.89. Fickian diffusion is a drug transport mechanism where structure relaxation is slower than diffusion. When relaxation exceeds diffusion, case-II transport occurs. Anomalous diffusion (non-Fickian transport) occurs when this drug release rate is close to structure relaxation [96,97,98,99].

(4) $$\log\frac{M_{\left(i-l\right)}}{M_{\infty }}=\log K+n\log\left(t-l\right)$$

where $M_{\infty }$ is the amount of drug at the equilibrium state, $M_{i}$ is the amount of drug released over time (t), K is the constant of incorporation of structural modifications and geometrical characteristics of the system (also considered the release velocity constant), n is the exponent of release (related to the drug release mechanism) in function of time t, and l is the latency time.

### 4.10. Scanning Electron Microscopy (SEM)

After seven days of drug release testing in PBS with a pH of 6.8, the Eudragit-based ISG remnants were washed with 200 mL of distilled water and then freeze-dried with the assistance of a freeze dryer (TriadTM Labconco, Kansas City, MO, USA). The following process took place after the remnants had been stored in a desiccator for one week. The dried Eudragit-based ISG remnants were then coated with gold before being examined using the SEM method and admitted of comparison with intact levofloxacin HCl powder. At an accelerating voltage of 15 kV, a scanning electron microscope (SEM) TESCAN MIRA3 from Brno-Kohoutovice, Czech Republic, was used to observe the surface and cross-sectional morphologies of a dried Eudragit-based ISG remnant from a developed ISG system.

### 4.11. In Vitro Degradation Test

The in vitro degradability of forming matrices was carried out by determining the mass loss of the system after the drug release test. The initial weight of the sample and that after the release test at 14 days were recorded and calculated (n = 3) as follows:

(5) $$\%\;\mathrm{mass}\;\mathrm{loss}=\left(\frac{W_{i}-W_{t}}{W_{i}}\right)\times 100$$

where

$W_{i}$*=* initial weight of the sample
$W_{t}$ = weight of remained sample at a specific time

### 4.12. X-ray Imaging and X-ray Tomographic Microscopy

The remnants of LL15M and LL20M were collected from drug release study, then washed with distilled water. They were dried together using a freeze dryer. The dried systems were kept in a desiccator for 7 days. The dried systems were scrutinized with X-ray imaging and X-ray tomographic microscopy at the X-ray tomographic microscopy (XTM) beamline, Synchrotron Light Research Institute (SLRI), Thailand. The following conditions were set: Generation of X-ray beam was archived by 2.2-T multipole wiggler at the 1.2-GeV Siam Photon Source facility (150 mA). The synchrotron radiation with X-ray tomographic microscopy (SRXTM) inspections was performed using a filtered polychromatic X-ray beam at a mean energy of 11.5 kV with a source-to-sample distance of 34 m. Detection system was equipped with a 200 µm thick scintillator (YAG: Ce, Crytur, Turnov, Czech Republic), lens-coupled X-ray microscope, and the CMOs camera (PCO edge 5.5, 2560 pixels, 16 bits) (Optique Peter, Lentilly, France). Isotropic voxel size of 3.61 µm was used. X-ray projection was normalized using the Octopus reconstruction (TESCAN). The tomographic volumes were achieved via Drishti software (National Computational Infrastructure’s VizLab). The porosity was determined using Octopus Analysis (TESCAN) [100].

### 4.13. Antimicrobial Activities

The antimicrobial activities of monopropylene glycol, drug-free, and levofloxacin HCl-loaded Eudragit-based ISG formulations were evaluated against standard microbes (*S. aureus* DMST 6532, *MRSA S. aureus* ATCC 4430, *S. aureus* ATCC 6532, and *S. aureus* ATCC 25923), *E. coli* ATCC 8739, *C. albicans* 10231, *P. gingivalis* and *A. actinomycetemcomitans*) using the agar diffusion assay (cylinder plate method). For this bioactivity test, bacteria inocula were incubated for 36 h in tryptic soy broth (TSB), whereas sabouraud dextrose broth (SDB) was employed for *C. albicans*. The turbidity of broth suspensions of organisms was calibrated using the 0.5 McFarland standard. Consequently, the attained broth suspensions of *S. aureus* DMST 6532, *S. aureus* ATCC 4430, *S. aureus* ATCC 6532, *S. aureus* ATCC 25923, and *E. coli* ATCC 8739 were swab-spread on the tryptic soy agar plates, whilst sheep blood agar and chocolate agar were used as media for anaerobic antimicrobial testing of *P. gingivalis* and *A. actinomycetemcomitans*, respectively. The inoculum of calibrated *C. albicans* was swab spread on sabouraud dextrose agar (SDA). The 200 microliters aliquot sample was filled into a sterilized cylindrical cup that was already placed on the surface of the swabbed agar before incubation at 37 °C for 24 h. In the case of antibacterial measurement against *P. gingivalis* and *A. actinomycetemcomitans,* the incubation was conducted in an anaerobic incubator (Forma Anaerobic System, Thermo Scientific, Ohio, USA) at 37 °C for 72 h. To indicate and compare the antimicrobial activities (n = 3), the diameter (mm) of the inhibition zone was measured individually with a ruler.

### 4.14. Fourier Transform Infrared (FTIR) Spectroscopy

In addition to that, an FTIR spectrophotometer was used in order to record the spectra of the samples. The sample was mixed with potassium bromide, and then it was compressed with a plunger and die in a hydraulic press (pressure of 5 tons). The obtained pellet was placed inside of an FTIR chamber after it had been mounted. The sample’s transmittance as a percentage was measured and recorded. In the range of 400–4000 cm^−1^, the spectra were collected at a resolution of 2 cm^−1^.^.^

### 4.15. Statistical Analysis

SPSS for Windows (version 11.5) was employed for the statistical analysis. The difference between the experiments was determined by using Student’s *t*-test. The results obtained were statistically significant because of the *p*-value, which was found to be less than 0.05. 

## Figures and Tables

**Table 1:** Regression coefficient (r^2^) value, msc, and diffusion exponent value (n) obtained from drug release profile of levofloxacin HCl-loaded Eudragit L-based ISG formulations fitting different mathematic equations.

Formula	Zero Order		First Order		Higuchi’s		Korsmeyer–PEPPAS				
	r^2^	msc	r^2^	msc	r^2^	msc	r^2^	msc	k ± SD	n ± SD	Release Mechanism
LLM10	0.8048	1.3742	0.8683	1.9759	0.9058	2.5144	0.9770	3.5961	3.344 ± 1.837	0.444 ± 0.050	Fickian diffusion
LLM15	0.7118	0.9164	0.8872	2.0854	0.8945	2.0844	0.9503	2.6867	2.603 ± 0.666	0.367 ± 0.028	Fickian diffusion
LLM20	0.7941	1.2387	0.9090	2.3402	0.9188	2.3419	0.9320	2.3732	1.856 ± 0.819	0.444 ± 0.055	Fickian diffusion

**Table 2:** Mass loss from in vitro degradation test of levofloxacin HCl-loaded Eudragit L-based ISG formulations. The superscripts (a–d) in the column represent a significant difference within the tested formulations (*p* < 0.05).

Formula	Weight Loss (%)				
	Day 1	Day 3	Day 5	Day 7	Day 14
LLM10	85.39 ± 0.32 ^a^	91.96 ± 0.98 ^b^	94.53 ± 1.46 ^c^	94.96 ± 1.21 ^d^	100.00 ± 0.00
LLM15	80.22 ± 2.90 ^a^	86.95 ± 1.97 ^b^	87.91 ± 0.54	92.57 ± 1.92	100.00 ± 0.00
LLM20	72.81 ± 1.85 ^a^	82.02 ± 1.46 ^b^	85.95 ± 1.98 ^c^	90.21 ± 1.80 ^d^	100.00 ± 0.00

**Table 3:** Clear zone diameter of monopropylene glycol, drug-free, and levofloxacin HCl-loaded Eudragit L-based ISG formulations against *S*. *aureus* (ATCC 6538, 6532, and 25923), methicillin-resistant *S*. *aureus* (*MRSA*) (*S*. *aureus* ATCC 4430), *E*. *coli* ATCC 8739, *C*. *albicans* ATCC 10231, *P*. *gingivalis* ATCC 33277, and *A*. *actinomycetemcomitans* ATCC 29522 (n = 3).

Formula	Clear Zone Diameter (mm.) Mean ± S.D.							
	*S*. *aureus* 6538	*S*. *aureus* 4430	*S*. *aureus* 6532	*S*. *aureus* 25923	*E*. *coli* 8739	*C*. *albicans* 10231	*P*. *gingivalis ATCC* 33277	*A*. *actinomycetemcomitans ATCC* 29522
MP	12.7 ± 0.5	13.0 ± 0.8	12.3 ± 0.5	10.3 ± 0.5	14.7 ± 0.5	18.7 ± 1.2	17.0 ± 2.2	26.3 ± 0.5
LM10	11.7 ± 0.5	10.7 ± 0.5	11.7 ± 0.5	9.8 ± 0.2	12.0 ± 0.8	16.7 ± 0.5	12.0 ± 0.8	23.7 ± 0.5
LM15	10.5 ± 0.4	10.7 ± 0.5	10.7 ± 0.5	**-**	12.0 ± 1.4	16.0 ± 0.8	15.0 ± 1.4	23.3 ± 0.5
LM20	10.3 ± 1.2	9.7 ± 0.5	11.3 ± 1.2	**-**	12.7 ± 1.2	15.0 ± 0.8	13.3 ± 1.2	22.3 ± 0.5
LVM	26.3 ± 0.9 ^a^	25.3 ± 0.9 ^b^	26.0 ± 0.8 ^c^	25.3 ± 0.5 ^d^	25.3 ± 0.9 ^e^	20.0 ± 1.6 ^f^	26.0 ± 0.8 ^g^	>40
LLM10	26.7 ± 0.5	23.3 ± 0.5	23.3 ± 1.2	23.3 ± 0.5	23.3 ± 0.9	15.3 ± 2.1	25.3 ± 1.2	>40
LLM15	25.3 ± 0.5	22.7 ± 0.5 ^b^	21.7 ± 0.5 ^c^	22.7 ± 1.2 ^d^	22.3 ± 0.5 ^e^	16.0 ± 0.8 ^f^	23.3 ± 1.2	>40
LLM20	24.3 ± 0.5 ^a^	21.3 ± 0.9 ^b^	20.3 ± 0.5 ^c^	20.7 ± 0.5 ^d^	22.0 ± 0.8 ^e^	14.7 ± 0.9 ^f^	21.3 ± 0.5 ^g^	>40

The superscripts (a–g) in the column represent a significant difference within the tested formulations (*p* < 0.05).

**Table 4:** Composition formula of 5–25% various Eudragit L, S in monopropylene glycol (A,B); and levofloxacin HCl-loaded 10–20% Eudragit L-based in monopropylene glycol.

A			
Formula	Amount (% *w*/*w*)		
	Eudragit L	MP	
LM5	5	95	
LM10	10	90	
LM15	15	85	
LM20	20	80	
LM25	25	75	
B			
Formula	Amount (% *w*/*w*)		
	Eudragit S	MP	
SM5	5	95	
SM10	10	90	
SM15	15	85	
SM20	20	80	
SM25	25	75	
C			
Formula	Amount (% *w*/*w*)		
	Levofloxacin HCl	Eudragit L	MP
LLM10	0.5	10	89.5
LLM15	0.5	15	84.5
LLM20	0.5	20	79.5
LVM	0.5	-	95.5

## Data Availability

The data presented in this study are available on request from the corresponding author.
